# Statistics of the Frequency of Cardiac and Pulmonary Complication in Acute Rheumatism

**Published:** 1846-04

**Authors:** P. N. Latham

**Affiliations:** Fellow of the Royal College of Physicians, &c.


*Statistics of the frequency of Cardiac and Pulmonary Complication in Acute Rheumatism.* By P. N. Latham, M. D., Fellow of the Royal College of Physicians, &c.—The following remarks embody the experience of Dr. Latham, as related by him in the recently published and most valuable work, before alluded to. He observes that between the years 1836 and 1840, both inclusive, he met with 136 cases of acute rheumatism, of which 75 were males, and 61 were females.

Of the 75 males, the heart was affected in 47, unaffected in 28. Of the 47 cases, the disease was confined to the endocardium in 30, to the pericardium in 3, and affected both simultaneously in 7. In 7 others, although the heart was undoubtedly affected, the exact seat of the disease was uncertain.

Of the whole number of males in whom the heart was thus affacted, 3 died, and in these 3 both pericardium and endocardium were inflamed.

Of the 61 females, the heart was affected in 43, and not so in 18. Of the 43 cases, the seat of the disease was the endocardium alone in 33; the pericardium alone in 4; and both membranes in 4; the exact seat of the disease was doubtful in 2. None of these females died.

The account therefore of both males and females will stand thus:

| | |
|---|---|
| No. of Cases | 136 |
| Heart exempt, - - - - - - | 46 |
| Heart affected, - - - - - | 90 |
| Seat of the disease in the heart: | |
| Endocardium alone in - - - - | 63 |
| Pericardium alone in - - - - | 7 |
| Endocardium and pericardium in - | 11 |
| Doubtful in - - - - - - | 9 |
| Deaths - - - - - - - | 3 |

Of the 63 patients who suffered from endocarditis, and who became convalescent, auscultation still revealed the fact, that after the inflammation had ceased, the membrane recovered complete integrity only in 17, and that it remained permanently uncured in 46. Of the 30 males, the endocardial murmur ceased entirely only in 8, while it remained as long as they continued under observation in 22. Of the 36 females, it ceased entirely only in 9; and remained in 24.

In the 136 cases which form the basis of this inquiry, the heart was inflamed in two-thirds of the whole; the lungs only in about 1 in 5. In these cases, 24 in number, the pulmonary affection was severe; they consisted of bronchitis 8 cases; of pneumonia 18; of both combined 9 cases; of pleurisy 2. Of these 24 cases, 2 proved fatal.

Of the 46 cases of acute rheumatism, in which there was no heart complication, the lungs were likewise free in 6, or in 1 in 9 cases. On the other hand, in the 90 cases in which the heart was affected, pulmonary disease also occurred in 1 in 5.—*Ranking's Abstract.*

